# Setting up a community-based cervical screening service in a low-income country: a pilot study from north-western Tanzania

**DOI:** 10.1007/s00038-017-0971-8

**Published:** 2017-04-19

**Authors:** Nestory Masalu, Patrizia Serra, Dino Amadori, Jackson Kahima, Charles Majinge, Joyce Rwehabura, Oriana Nanni, Sara Bravaccini, Maurizio Puccetti, Rosario Tumino, Lauro Bucchi

**Affiliations:** 1 Oncology Unit, Bugando Medical Centre (BMC), Mwanza, Tanzania; 2 Unit of Biostatistics and Clinical Trials, Istituto Scientifico Romagnolo per lo Studio e la Cura dei Tumori (IRST) IRCCS, Meldola, Italy; 3 Department of Medical Oncology, Istituto Scientifico Romagnolo per lo Studio e la Cura dei Tumori (IRST) IRCCS, Meldola, Italy; 4 Pathology Unit, Bugando Medical Centre (BMC), Mwanza, Tanzania; 5 Dodoma Christian Medical Centre (DCMC), Dodoma, Tanzania; 6 Biosciences Laboratory, Istituto Scientifico Romagnolo per lo Studio e la Cura dei Tumori (IRST) IRCCS, Meldola, Italy; 7 Pathology Unit, S. Maria delle Croci Hospital, Ravenna, Italy; 8 Cancer Registry and Histopathology Unit, Civic MP Arezzo Hospital, ASP Ragusa, Ragusa, Italy; 9 Romagna Cancer Registry, Istituto Scientifico Romagnolo per lo Studio e la Cura dei Tumori (IRST) IRCCS, Meldola, Italy

**Keywords:** Sub-Saharan Africa, Cervical cancer, Prevalence, Screening

## Abstract

**Objectives:**

To report the results of a pilot study for a service for cervical cancer screening and diagnosis in north-western Tanzania.

**Methods:**

The pilot study was launched in 2012 after a community-level information campaign. Women aged 15–64 years were encouraged to attend the district health centres. Attendees were offered a conventional Pap smear and a visual inspection of the cervix with acetic acid (VIA).

**Results:**

The first 2500 women were evaluated. A total of 164 women (detection rate 70.0/1000) were diagnosed with high-grade cervical intraepithelial neoplasia and invasive cervical cancer. The performance of VIA was comparable to that of Pap smear. The district of residence, a history of untreated sexually transmitted disease, an HIV-negative status (inverse association), and parity were independently associated with the detected prevalence of disease. The probability of invasive versus preinvasive disease was lower in HIV-positive women and in women practicing breast self-examination.

**Conclusions:**

The diagnostic procedure had an acceptable level of quality. Factors associated with the detected prevalence of disease will allow for a more targeted promotion of the service. Cervical screening should be coordinated with sexually transmitted disease and HIV infection control activities.

## Introduction

In sub-Saharan Africa cervical cancer is the leading cause of cancer deaths, with an incidence of disease among the highest worldwide and one of the lowest 5-year survival rates (Jemal et al. 2012; Parkin et al. 2008; Sankaranarayanan et al. 2010; Soerjomataram et al. 2012). The human and social costs are enormous (Fokom-Domgue et al. 2014) and the burden of disease is expected to worsen. It is increasingly perceived that cervical cancer in this region must be given the same priority as human immunodeficiency virus (HIV) infection, malaria, and tuberculosis (Anorlu 2008). The major challenges to this come from the low level of education, limited human and financial resources, and poor professional/technical skills (Anorlu 2008; Fokom-Domgue et al. 2014; Tekinturhan et al. 2013).

For these reasons, the provision of cervical cancer prevention services is far below the needs (Chigbu et al. 2015). In particular, cervical screening coverage ranges locally from near 0% to no more than 15–20% (Louie et al. 2009). There are few population-based screening activities and none of sub-Saharan African countries has extended the provision of the service (Louie et al. 2009; Mvundura and Tsu 2014).

These are also the consequences of the fact that the main established screening techniques, i.e., cervical cytology and human papillomavirus testing, are cost-prohibitive in most parts of the region and are not supportable by the existing health care infrastructure (Denny et al. 2013; Jemal et al. 2012; Saxena et al. 2012). An alternative approach is visual inspection with acetic acid (VIA) and visual inspection with Lugol’s iodine (VILI) (Sankaranarayanan et al. 2003). Cervical cancer control interventions using these techniques are particularly suitable in low-resource settings (Ginsberg et al. 2012; Shastri et al. 2005), provided that adequate information is supplied to the population. In addition to having an acceptable level of sensitivity, visual screening techniques can be immediately followed by treatment with cryotherapy in a single visit (Saxena et al. 2012). Moreover, the introduction of visual screening techniques on a large scale may help to develop the infrastructure which, in turn, will facilitate the adoption of more affordable human papillomavirus testing technologies in the future (Sankaranarayanan et al. 2013).

In this perspective, there is a need to conduct health service research studies on feasible VIA/VILI screening models that can be generalised to low-resource environments (Hanna and Kangolle 2010; Morhason-Bello et al. 2013; PATH 2004). The data for the present study were collected during the one-year pilot phase of a newly implemented service for cervical cancer and breast cancer screening and diagnosis in Tanzania. The study rationale was twofold: first, we considered it necessary to obtain a field confirmation of the diagnostic performance of VIA as reported in research settings (Sankaranarayanan et al. 2003); and, second, we aimed to determine the demographic and health-related characteristics of patients that identify subsets of the population with higher prevalence of disease, particularly invasive disease, in order to target them with greater screening intensity (additional and tailored advertising, shorter interscreening intervals, etc.).

## Methods

### Setting

The pilot study was part of the Mwanza Cancer Project, a cooperative Italian-Tanzanian initiative to establish a comprehensive regional cancer centre in north-western Tanzania. The project is described elsewhere (Amadori et al. 2016).

The target area included eight districts in the Mwanza and Mara Regions (Victoria Lake Zone), with a total land surface of 42,000 km^2^ and a population of about 5,200,000 inhabitants. The pilot study was developed in 2011. With respect to cervical disease, its purposes were: (1) to evaluate the feasibility of, and the community involvement in, a scheme for cervical screening and diagnosis designed for the rural districts; (2) to build local skills for screening and diagnosis of cervical disease; (3) to develop effective information techniques; (4) to facilitate the implementation of a sustainable region-wide programme; and (5) to raise disease awareness in the community (PATH 2004).

### Endpoints

The endpoints of the current study included the following: (1) the positivity rate, the rate of loss to diagnostic follow-up, the disease detection rate, and the positive predictive value of VIA relative to Pap testing; (2) the association between demographic, socioeconomic, and health-related characteristics of attending women and their likelihood of being diagnosed with high-grade cervical intraepithelial neoplasia (CIN) or invasive cervical carcinoma; and (3) the association between the same characteristics and the likelihood of affected patients having invasive versus pre-invasive disease.

### Organisation

The programme was conducted in the health centres, i.e., the intermediate health posts in the district health system between dispensaries and the district hospital. In the rural districts, 6–7 health centres per district provide primary healthcare and outpatient services to an average catchment area of 9–10 villages and a population of about 60,000 inhabitants.

Thirty-two nurses underwent a 6-day practical/theoretical training programme in smear taking and VIA at their health centre of employment. In February 2012, a community-level information campaign was conducted across the target area using leaflets as the main medium. The campaign was partnered by general practitioners, village authorities, local church leaders, and primary school teachers. Women between 15 and 64 years of age (about 650,000 according to the 2012 National Census) were encouraged to attend the service. In each health centre, two 4-h sessions of visits per day for one or two days were conducted by multidisciplinary teams comprising the local general practitioner, an oncologist, a nurse, and a data collector. Although the World Health Organization screening recommendations concentrate on women aged 25 years or older (World Health Organization 2006), we included sexually active women aged 15–24 years in the target age range for research purposes, i.e. to assess their prevalence of disease at screening, which has not yet been investigated in this region.

### Screening procedure

Women who presented were interviewed using a structured questionnaire. They were offered Pap testing, VIA, and clinical breast examination, free of charge. During the visit, women were evaluated for clinical signs of gynaecologic diseases, and an HIV test was also offered. Women reporting previous positive HIV test results were accepted for screening (Khozaim et al. 2014).

Pap smear and VIA were performed with standard techniques (Sankaranarayanan et al. 2003). Women with positive VIA results were offered immediate treatment with cryotherapy if the lesion met accepted criteria (Shastri et al. 2005). Women with positive VIA results who were ineligible for cryotherapy were referred to the Bugando Medical Centre (BMC) for colposcopy. Early repeat testing was recommended to women wishing to discuss the results of VIA with their family members.

Pap smears were transferred to the BMC for evaluation by local pathologists. The cytology reports were returned to the health centres within 2–3 weeks and released to women by nurses. In the case of a previous positive VIA result followed by cryotherapy, women with abnormal Pap testing results were not recommended for further follow-up. In the event of a previous negative VIA result, the cytology cut-off for referral to the BMC included atypical squamous cells of undetermined significance as well as abnormal cells not otherwise specified.

Colposcopy examination at the BMC was preceded by a repeat VIA. If an abnormal transformation zone was visualized, a punch biopsy was taken. Women with histologically confirmed high-grade CIN and invasive carcinoma were treated by a loop electrosurgical excision procedure or cold knife conization or hysterectomy after appropriate evaluation.

### Data analysis

We evaluated data from the first 2500 consecutive women who were seen between 23 May 2012 and 31 May 2013. We excluded 117 women who accepted only the clinical breast examination, 29 women who received only Pap testing, 7 women with clinically overt cervical cancer, and 5 women whose data were lost for the greater part. The total number of eligible women was 2342.

The cytological and histological diagnoses reported as high-grade squamous intraepithelial lesion were reclassified as high-grade CIN. Histologically confirmed high-grade CIN and invasive cervical carcinoma were assumed to be the target diseases.

To calculate the detection rate of disease, the sensitivity and the positive predictive value of VIA and Pap testing, the number of diseases detected with each test was adjusted for the rate of loss to follow-up. The denominator of sensitivity was the adjusted number of target diseases detected on either test or both tests. Women lost to follow-up were assumed to include those immediately treated with cryotherapy (without histologic diagnosis) as well as those who did not present for colposcopic assessment. The performance measures of VIA and Pap testing were compared by calculating their ratio with 95% confidence interval (CI) (Miettinen 1976).

Differences in proportions were tested for significance with the *χ* ^2^ tests for heterogeneity and trend. Differences in distribution were tested with the nonparametric Kruskal–Wallis test and Mann–Whitney test. The level of statistical significance was set at *p* < 0.05.

The odds ratio (OR), with 95% CI, of being diagnosed with the target disease and the OR for an affected woman of having invasive cancer (late disease stage) versus high-grade CIN (early stage) were estimated from backward stepwise multiple logistic regression models. The variables were removed if the likelihood ratio statistic based on the maximum likelihood estimates had a probability >0.1.

## Results

### Characteristics of attending women

**Fig. 1:**
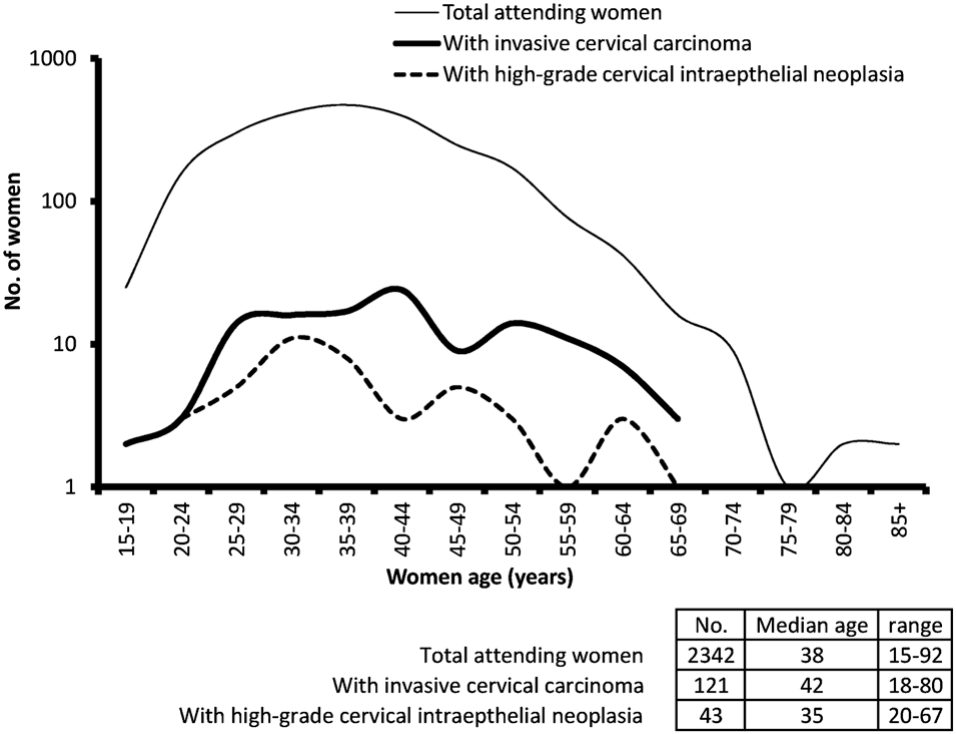
Age distribution of the 2342 eligible women (Tanzania, 2012–2013)

The age distribution of the 2342 eligible women is shown in Fig. 1 (thin curve). Table 1 shows their distribution according to categorical study variables. Housewives and unemployed women accounted for less than 20% of the study group. A little less than 50% of women reported using some type of contraception. There was a total of 572 (24.4%) women with previous diagnosis of sexually transmitted disease (STD), and 192 (8.2%) HIV-positive women. About 7% of women reported practicing breast self-examination.

**Table 1:** Characteristics of study women (total number of women 2342) (Tanzania, 2012–2013)

Characteristic	No. (%)
Demographics^a^	
District of residence	
Bukumbi	227 (9.7)
Kibara	249 (10.6)
Makoko	288 (12.3)
Musoma	266 (11.4)
Sengerema	380 (16.2)
Shinianga	532 (22.7)
Ukerewe	400 (17.1)
Marital status	
Married	1604 (68.5)
Other and unknown	738 (31.5)
Parity (tertiles)	
0–2	775 (33.1)
3–4	753 (32.2)
5–18	814 (34.8)
Socio-economic status	
Occupation	
Housewife, unemployed	415 (17.7)
Peasant, breeder	834 (35.6)
Craftswoman, dealer	512 (21.9)
Teacher, student	376 (16.1)
Other and unknown	205 (8.8)
Health and health behaviour	
Contraceptive use and type	
No and unknown	1301 (55.6)
Condom, IUD, loop	121 (5.2)
Oral contraceptives	436 (18.6)
Depoprovera, implant	408 (17.4)
Tube ligation	76 (3.2)
History of STD	
No and unknown	1770 (75.6)
Yes, untreated	168 (7.2)
Yes, treated	404 (17.3)
HIV testing	
No and unknown	678 (28.9)
Yes, negative	1472 (62.9)
Yes, positive	192 (8.2)
BSE practice	
No	2187 (93.4)
Yes	155 (6.6)

*IUD* intrauterine device, *STD* sexually transmitted disease, *HIV* human immunodeficiency virus, *BSE* breast self-examination

^a^See Fig. 1 for women’s age

### VIA and Pap testing results

Three hundred and two (12.9%) of the attending women tested positive on either VIA or Pap smear or both. Of these, 34 (11.3%) underwent immediate cryotherapy without histologic confirmation of disease, 26 (8.6%) were recommended for early repeat testing, 53 (17.6%) did not present for colposcopic assessment, 25 (8.3%) had a negative colposcopy and/or biopsy, and 164 (54.3%) were diagnosed with high-grade CIN and invasive cervical carcinoma with a detection rate of 70.0/1000 screenees. The overall rate of loss to follow-up was 28.8%.

Out of the 164 screen-detected women, 121 (73.8%) were diagnosed with invasive cervical cancer. In Fig. 1, the age distribution of women with pre-invasive (dashed curve) and invasive disease (bold curve) is shown. Women with invasive disease were older, but at a borderline level of significance (*p* = 0.071). The detection rate of high-grade CIN and invasive cervical carcinoma among women aged 15–24 years (*n* = 181) was 44.2/1000 screenees.

The results of VIA were compared with those obtained with Pap testing over the 1360 women who accepted to undergo both tests (Table 2). A total of 183 women tested positive either on VIA (*n* = 43) or Pap testing (*n* = 75) or both (*n* = 65). These groups were not significantly different in age (*p* = 0.39). VIA had an approximately 20% lower positivity rate and more than a twofold higher rate of loss to follow-up. Both were major factors in determining a considerably lower crude detection of disease. After adjustment for the rate of loss to follow-up, detection rate and sensitivity were approximately 10% poorer on VIA than on Pap testing, and no significant or only weakly significant differences were observed. In absolute terms, the sensitivity of both techniques was around 70%. Due to a lower positivity rate, VIA exhibited a greater positive predictive value, albeit with a low level of significance.

**Table 2:** Performance measures of visual inspection with acetic acid relative to Pap testing (total number of women 1360) (Tanzania, 2012–2013)

Measure	VIA	Pap testing	VIA:Pap testing ratio (95% CI)
Positivity rate (%)	108/1360 (7.9)	140/1360 (10.3)	0.77 (0.61–0.98)
Rate of loss to diagnostic follow-up^a^ (%)	45/108 (41.7)	26/140 (18.6)	2.24 (1.51–3.34)
Crude DR of disease^b^ (per 1000)	53/1360 (3.9)	82/1360 (6.0)	0.65 (0.46–0.90)
Adjusted DR of disease^b,c^ (per 1000)	90.9/1360 (6.7)	100.7/1360 (7.4)	0.90 (0.69–1.19)
Adjusted sensitivity rate^c^ (%)	89.0/134.7 (66.1)	101.6/134.7 (75.4)	0.88 (0.75–1.02)
Adjusted positive predictive value^c^ (%)	89.0/108 (82.4)	101.6/140 (72.6)	1.14 (0.99–1.30)

*VIA* visual inspection with acetic acid, *DR* detection rate, *CI* confidence interval

^a^Women lost to diagnostic follow-up include those who were immediately treated with cryotherapy as well as those who did not present for colposcopic assessment

^b^Target disease included high-grade cervical intraepithelial neoplasia and invasive cervical cancer

^c^Adjusted for the rate of loss to diagnostic follow-up

### Disease prevalence

Table 3 shows that factors significantly associated with the detected prevalence of disease in multiple logistic regression analysis included: district of residence, parity, a history of untreated STD, and a negative HIV test result. The latter was associated with a decreased probability of disease detection.

**Table 3:** Significant multivariate determinants of detected prevalence of disease (total number of women 2342) (Tanzania, 2012–2013)

Determinant	Total no. of women	Percent with disease^a^	*p* value	Unadjusted OR (95% CI)	Adjusted OR^b^ (95% CI)
District of residence			0.000		
Bukumbi	227	10.1		1.00 (reference cat.)	1.00 (reference cat.)
Kibara	249	4.0		0.37 (0.17–0.80)	0.35 (0.16–0.75)
Makoko	288	1.7		0.16 (0.06–0.42)	0.16 (0.06–0.42)
Musoma	266	19.5		2.15 (1.27–3.65)	2.16 (1.25–3.76)
Sengerema	380	2.9		0.26 (0.13–0.55)	0.19 (0.09–0.42)
Shinianga	532	10.7		1.06 (0.64–1.77)	1.17 (0.70–1.97)
Ukerewe	400	1.5		0.13 (0.05–0.34)	0.16 (0.06–0.40)
Parity (tertiles)			0.002^c^		
0–2	775	5.3		1.00 (reference cat.)	1.00 (reference cat.)
3–4	753	6.2		1.19 (0.77–1.83)	1.07 (0.68–1.70)
5–18	814	9.3		1.84 (1.24–2.73)	1.87 (1.17–2.99)
History of STD			0.008		
No and unknown	1770	7.2		1.00 (reference cat.)	1.00 (reference cat.)
Yes, untreated	168	11.3		1.64 (0.98–2.72)	1.99 (1.07–3.71)
Yes, treated	404	4.2		0.56 (0.34–0.95)	0.75 (0.43–1.31)
HIV testing			0.000		
No and unknown	678	8.1		1.00 (reference cat.)	1.00 (reference cat.)
Yes, negative	1472	5.6		0.68 (0.47–0.96)	0.71 (0.46–1.00)
Yes, positive	192	13.5		1.77 (1.08–2.92)	1.51 (0.84–2.71)

*OR* odds ratio, *CI* confidence interval, *STD* sexually transmitted disease, HIV human immunodeficiency virus

^a^Including high-grade cervical intraepithelial neoplasia and invasive cervical cancer

^b^Woman’s age (continuous variable) was forced into the model (in-block inclusion of variables). Occupation, marital status, contraceptive use and type, and breast self-examination practice were removed as nonsignificantly contributing to its likelihood (*p* > 0.1)

^c^Test for trend

### Tumour stage

Table 4 shows that the univariate probability for screen-detected patients to have an invasive cervical cancer versus a high-grade CIN varied significantly between districts, and was lower for HIV-positive women and for the small group of women practicing breast self-examination. These two variables were mutually adjusted in a backward stepwise multiple logistic regression model. HIV-positive status and breast self-examination practice yielded an OR of invasive disease detection of 0.22 (95% CI 0.08–0.63) and 0.06 (95% CI 0.01–0.33), respectively. The district of residence was not entered in the model because of cells with zero values.

**Table 4:** Significant univariate determinants of the probability for screen-detected women to have an invasive cervical cancer versus a high-grade cervical intraepithelial neoplasia (total number of women 164) (Tanzania, 2012–2013)

Determinant	Total no. of women	Percent with invasive cancer	*p* value
District of residence			0.000
Bukumbi	23	78.3	
Kibara	10	30.0	
Makoko	5	100.0	
Musoma	52	88.5	
Sengerema	11	100.0	
Shinianga	57	64.9	
Ukerewe	6	16.7	
HIV testing			0.036
No and unknown	55	80.0	
Yes, negative	83	75.9	
Yes, positive	26	53.8	
Breast self-examination			0.000
No	155	76.8	
Yes	9	22.2	

Woman’s age (continuous variable), marital status, parity, occupation, contraceptive use and type, and history of sexually transmitted disease were nonsignificantly associated with the dependent variable (*p* > 0.05)

*HIV* human immunodeficiency virus

## Discussion

The pilot study reported in this article was entirely self-managed by local staff after adequate training. This reflects the vision of the Mwanza Cancer Project, which aims to create sustainable cancer care services focused on local priorities. As for several other services, the pilot study was enabled by the establishment of a pathology service and an inpatient and outpatient medical oncology unit at the BMC, with an essential labour force (Amadori et al. 2016).

In sub-Saharan Africa, decentralisation is a key facilitator for initiating regional programmes for cervical screening (McCree et al. 2015). In our data, however, the high occupational profile of attending women suggested that socioeconomic selection factors are frustrating, at least in part, our efforts to target the rural population. More knowledge is needed about barriers to participation in cervical screening in this part of the world (Perng et al. 2013). Interventions aimed at improving women’s participation have mostly been studied in affluent countries and may not be either as effective or as feasible in developing countries (Everett et al. 2011). The level of women’s attendance to referral for assessment and treatment also needs consideration. The rate of loss to diagnostic follow-up was as high as 28.8%, although this was, in part, inevitably due to the use of immediate cryotherapy.

Regarding the performance of screening tests, the detection rate and sensitivity rate of VIA were fairly comparable to those of Pap testing after adjustment for the higher rate of loss to follow-up. We can confirm that the value of visual techniques is in the same order of magnitude as that of cervical cytology (Sankaranarayanan et al. 2003; Saxena et al. 2012; Shastri et al. 2005).

At screening, the detected prevalence of disease was approximately twofold greater than expected on the basis of comparable literature data from low-middle income countries (Sankaranarayanan et al. 2003). This primarily reflects the higher risk of disease among sub-Saharan women. However, we also believe that the sample of women attending the programme was not representative of the general population. Probably, some women with clinically overt cancer (not eligible for the current study) were not identified and excluded from the analysis, as is suggested by the fact that the number of detected invasive diseases exceeded that of pre-invasive diseases. Owing to this high prevalence, both screening tests attained uncommon levels of detection rate and positive predictive value in spite of their moderate sensitivity.

The identification of independent determinants of disease prevalence at screening represented the most interesting result of this study. These factors include the district of residence, a history of untreated STD, an HIV-negative status (associated with a decreased probability of disease detection), and parity. The latter three are in accordance with the epidemiology of cervical disease, whereas the effect of the district of residence depends on differences in the prevalence of risk factors and of previous attendance at a gynaecologic clinic. By implication, the Musoma, Bukumbi, and Shinyanga districts will have to be targeted with greater screening intensity. A disappointing finding is that none of these determinants is known to be favourably associated with the uptake of cervical screening in sub-Saharan Africa (as is the case for contraceptive use, for example) (Thulaseedharan et al. 2015).

The excess detected prevalence of disease observed among women reporting a history of untreated STD deserves more attention as it has been suggested that cervical screening activities in sub-Saharan Africa should be integrated with other health and social services (Hanna and Kangolle 2010), in particular with STD control practices (Osingada et al. 2015). Given the dual burden of cervical cancer and HIV in the region, the coordination of cervical screening with HIV infection control programmes could be an even more cost-effective policy (Menéndez et al. 2010). On the one hand, women who receive antiretroviral therapy can be observed more regularly and can receive the continuity of care needed for an effective cancer screening. On the other hand, cervical screening of HIV-infected women treated with antiretroviral drugs may have a greater impact on their life expectancy because of the lower competing mortality associated with other causes (Jaquet et al. 2014).

It is worth noting that we found a decreased detected prevalence of disease among women reporting a negative HIV test result but failed to confirm the opposite among HIV-positive women. Since cervical lesions are less common among HIV-infected women without immune deficiency, it could be hypothesised that antiretroviral therapy and immunological recovery have lowered the risk of both CIN and cervical cancer. Of course, the inclusion of screening programmes in the surveillance of HIV-infected women living in sub-Saharan Africa remains a priority (Franceschi and Jaffe 2007; Menéndez et al. 2010).

The analysis of the likelihood of invasive disease at diagnosis, albeit limited by the relatively small sample of screen-detected lesions, provided suggestive results. The likelihood was less for HIV-positive women and for women practicing breast self-examination. We have no data to hypothesise that an HIV-positive status was associated with an increased frequency of medical checks and, thus, with a higher prevalence of previous gynaecological examinations or more recent examinations. Our finding warrants further investigation. To this end, we suggest a closer collaboration between cervical screening and HIV infection control activities in the region. This is one of the key strategies proposed to improve cervical cancer control in sub-Saharan Africa (Amadori et al. 2016; Hanna and Kangolle 2010; Menéndez et al. 2010).

In conclusion, the results of the pilot study may be summarised as follows: the feasibility of the programme has been demonstrated; a permanent screening workforce of multidisciplinary teams has been trained; the diagnostic procedure has shown an acceptable level of quality; a set of communication tools designed to increase the awareness of cervical cancer in the community has been developed; and the analysis of data has confirmed that coordination of cervical screening activities with HIV infection and STD control activities is an appropriate policy. The two main adverse findings were the limited accessibility of the service to lower-class women living in rural areas and the high rate of loss to follow-up. The pilot study has become a permanent programme.
